# High-Dimensional Coexistence of Temperate Tree Species: Functional Traits, Demographic Rates, Life-History Stages, and Their Physical Context

**DOI:** 10.1371/journal.pone.0016253

**Published:** 2011-01-31

**Authors:** Sean M. McMahon, Charlotte J. E. Metcalf, Christopher W. Woodall

**Affiliations:** 1 Smithsonian Tropical Research Institute, Center for Tropical Forest Science, Smithsonian Environmental Research Center, Edgewater, Maryland, United States of America; 2 Department of Zoology, Oxford University, Oxford, United Kingdom; 3 Forest Inventory and Analysis, U.S. Forest Service, St. Paul, Minnesota, United States of America; National Institute of Water & Atmospheric Research, New Zealand

## Abstract

Theoretical models indicate that trade-offs between growth and survival strategies of tree species can lead to coexistence across life history stages (ontogeny) and physical conditions experienced by individuals. There exist predicted physiological mechanisms regulating these trade-offs, such as an investment in leaf characters that may increase survival in stressful environments at the expense of investment in bole or root growth. Confirming these mechanisms, however, requires that potential environmental, ontogenetic, and trait influences are analyzed together. Here, we infer growth and mortality of tree species given size, site, and light characteristics from forest inventory data from Wisconsin to test hypotheses about growth-survival trade-offs given species functional trait values under different ontogenetic and environmental states. A series of regression analyses including traits and rates their interactions with environmental and ontogenetic stages supported the relationships between traits and vital rates expected from the expectations from tree physiology. A combined model including interactions between all variables indicated that relationships between demographic rates and functional traits supports growth-survival trade-offs and their differences across species in high-dimensional niche space. The combined model explained 65% of the variation in tree growth and supports a concept of community coexistence similar to Hutchinson's n-dimensional hypervolume and not a low-dimensional niche model or neutral model.

## Introduction

A number of potential mechanisms have been proposed to explain how species can coexist in diverse forests. These include unique species responses to environmental heterogeneity [1], ontogenetic shifts in demographic rates (specifically growth and survival responses of small trees versus large trees) [2], [3], as well as trade-offs (such as growth-survival trade-offs or shade tolerance and light-tolerance [4]). Another important development in the ecological literature shows that functional traits provide evidence that the allocation of resources to physiologically distinct leaf characteristics, wood density, and seed mass might lead to the coexistence of many species under similar conditions [5], [6]. Quantifying the relationship between functional traits and demographic rates (such a growth and survival) can therefore be used to link species physiological differences to the potential for coexistence [6], [7]. If functional traits confer advantages to different species under different environmental [8], [9] or ontogenetic [10] conditions, this could help explain the high diversity of coexisting forest tree species.

Coexistence mediated through demographic rates has been central to theories of coexistence in diverse forests, most commonly through a trade-off between growth and survival [11] (species that grow slowly tend to survive longer, while species that grow fast would have higher mortality [11]). This might operate in the context of forest succession. After a disturbance a fast growing species can preferentially acquire light, while slow-growing species that can survive in the understory can supplant high-mortality species as they die [12]. Functional traits, such as the leaf mass per area (LMA), seed mass, and wood density, tend to be organized along this trade-off and have often been implicit in studies of growth-survival trade-offs relating to gap-phase dynamics [13], [14]. Faster growing trees often have lower LMA, smaller seed mass, and low wood density [6], but fast-growing species, and the functional traits associated with them, are commonly shade intolerant (i.e., require high-light environments for growth and survival) [13]. Conversely, shade tolerance may be associated with dense wood, large seeds, and high LMA, which could promote a capacity to survive water stress, frost, fire, or herbivore attacks [11], but this investment in tolerance limits investment in growth.

Several studies have linked functional traits and the environment [15]–[17], others demographic rates and functional traits [6], [7]. Studies have also linked demographic rates and ontogenetic and environmental variables [18]–[20]. Combining environmental, ontogenetic, and trait variables in one analysis can test whether traits can lead to coexistence and determine the important links between the environment and vital rates, mediated by traits, that drive coexistence. These mechanistic links are critical when considering how climate change might affect the future patterns of forest tree species [21].

An example that can illustrate the importance, and potential complexity, of trait-mediated patterns of population change would be the common trade-offs expected from leaves [22]. Thick leaves (high leaf mass per area [LMA]), reduce photosynthesis, and therefore lead to slower growth. Thicker leaves, however, are better at water retention under drier conditions. A trade-off between high-growth and survival under stressful conditions driving coexistence, however, is entirely dependent on there being spatial or temporal variation in the hydrological environment at a scale that differentially influences vital rates, and thus long-term population trends. If soils in an area are generally dry, one would expect thick-leaved trees to persist (also termed ‘environmental filtering’ [5]), under moist conditions, only thin-, or broad-leaved trees to persist. Temporally, if drought follows a frequency and dependency that benefits high, and then low LMA species, coexistence will follow the intermediate disturbance hypothesis [23]. Explaining these relationships for multiple species is more difficult. Yet, one physiologically, the relationship between LMA, soil moisture, and vital rates can be more critical for small trees, as large trees tend to have greater access to water through mature root-systems and light because of canopy status (although exceptions exist, which further add to the persistence of multiple species in a local forest). It is exactly through the conditional dependencies then that these trait-rate-environment trade-offs can explain not simply the coexistence of two leaf types, but using only LMA, soil moisture, and tree ontogeny, the coexistence of many more species. How do small trees in high-light environments grow? Does this growth affect mortality? Does soil moisture remain important? This thought experiment in the complexity of coexistence is neither new, nor novel (e.g., [24], [25], [26]). It is difficult to test because of the need for a great deal of observational data, the statistical tools to analyze those data.

In this study, we quantify the relationship between demographic rates and functional traits in 41 tree species in Wisconsin across 505 different plots, incorporating the role of ontogenetic shifts and environmental context. Functional traits considered here include leaf mass per area (LMA), a measure of wood specific density, and seed size. The environmental variables include light, stand age, and slope with a northeastern aspect. The two latter variables act as proxies for site characteristics including micro-climate and soil variables. For development of the growth estimates, we employ a Bayesian approach because varying census periods in these data are difficult to accommodate with maximum likelihood approaches. We then use posteriors samples of the growth model to fit a model of the effects of size on survival. Using posterior means derived from these Bayesian models we build a broader likelihood model to predict growth and survival of all species under a range of environmental and ontogenetic conditions, to identify relationships between traits and demography, and quantify the role of environmental and ontogenetic context.

The central goals of this analysis include determining 1) whether the growth-survival trade-off predicted by theory is found in a broad range of plots in a temperate forest, 2) whether growth and survival relate to functional traits across forest plots, 3) how these traits-vital rate relationships depend on environmental conditions and ontogenetic stage, and 4) the extent to which all variables combined can explain growth patterns in temperate forest trees. In the following section, we introduce the demographic data and describe the models used for analysis, followed by a description of analytical results and a discussion of their implications.

## Methods

The overall approach used forest inventory data from the U.S. Department of Agriculture, Forest Service's Forest Inventory and Analysis (FIA) program to infer parameters for species-specific models of growth and survival. We then tested relationships between key demographic features and functional traits given the environment by predicting demographic rates from parameters in different environmental contexts.

### Data sources

Three types of information were obtained from the FIA records (http://fia.fs.fed.us/) across 505 plots in Wisconsin, incorporating ∼31,000 individuals. The data include: i) diameter at breast height in three years: 1983, 1996, and a date between 1999 & 2004, denoted *D_i_*_,*j*,*t*_ the diameter of individual *i* at time *t* in plot *j*; ii) survival status (alive, dead), *s_i_*_,*t*_, and iii) an index of the light environment for each tree, *L_i,t_* taken on a categorical scale from one to five, where the categories indicate “Open Grown”, “Dominant Trees”, “Co-dominant Trees”, “Intermediate Trees”, and “Overtopped”. Plot level characteristics include a measure of stand age, either estimated by researchers or obtained from coring information, and an estimate of stand aspect, taken as the direction of slope of the subplot to the nearest degree determined along the direction of slope with North set as 360. For further details regarding FIA's sampling design and methods see Bechtold and Patterson [27]. We obtained estimates of Leaf Mass per Area (LMA) from Wright *et al.* [22]. Many other leaf traits are available, but as leaf traits covary, we focused on LMA. We obtained estimates of seed size using the data-base provided by Kew Gardens (http://data.kew.org/sid/sidsearch.html). The World Agroforestry Centre [28] and Loehle [29] were used to obtain estimates of wood density. Individuals in the genus *Crataegeus* were not identified to species in the FIA data-base and constitute a shrub so were removed from the analysis. No estimates were available for *Acer saccharin* LMA or seed size, so we used the average for all values provided across this genus for seed size, LMA and wood density. *Quercus bicolor* and *Carpinus carolinia* had outlier survival rates due to small sample size and were excluded from analyses.

Many studies that explore functional traits employ phylogenetic independent contrasts (PICS) to account for correlations between related species in trait values. To consider phylogenetic correlation in the data-set we provide PIC correlations between functional traits and demographic rates in a range of environmental conditions (Supplemental material). We found similar trends when phylogenetic correlation was taken into account, but as our focus was on interactions and ANCOVAs with environmental factors, which cannot be accommodated by a PIC analysis, we performed all of our regression analyses directly on the species data.

### Bayesian modeling framework

Below we briefly define the rationale behind the probability models used for each of the components of our model, including observation models (e.g. observation error in growth), process models (e.g. growth), and the priors for parameters.

### Modeling uncertainty in diameter measurements and mortality with observation error and uneven census intervals

A first challenge to modeling this dataset is that there may be errors in measurements leading to unlikely large negative growth increments. To allow for measurement error, as well as shrink and swell of the bole, we define a model to describe error in the measured diameter values. We assume that the observed diameter in cm of individual *i* at time *t* in plot *j*, 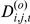, (where “observation” is indicated by the superscript ^(*o*)^) is normally distributed around the “true” diameter with a variance *w* that captures measurement error, e.g., 
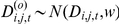
. We fit the growth models to this distribution of inferred “true” diameter values. The next challenge to inferring yearly growth increments in this dataset is that census intervals are uneven. We avoid a bias due to census intervals by estimating how variance accumulates in growth over the gaps lacking census data. Denoting *D_i_*_,j,*t*_ as diameter in cm of individual *i* at time *t* in plot *j*, and Δ the time between censuses, we define the increment between diameter at *t* and diameter at *t*+Δ by ϕ. If the mean growth increment in one year for individual *i* in plot *j* in year *t* is φ, and the variance of such yearly growth increments is *σ* ^2^. Multiplying the variance by Δ, the time between censuses, captures a standard assumption of white noise in yearly increments. This approach is described in detail in Metcalf et al. [30].

Having established the density function used for modeling growth *μ*, we defined covariates and associated parameters we wish to estimate for the growth process as
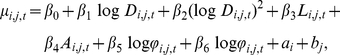
(1)


where the *i* index refers to individual, the *j* index refers to the plot number, and the *t* index refers to the time step, *D* is diameter in cm, *L* is the light index, *A* is estimated stand age, ***β*** is a vector of parameters, *a_i_* is a random individual effect, and *b_j_* is a random plot effect. The individual effect value accounts for variation over and above that defined by covariates, and that remains consistent during individual's lifetime [31] (see Appendix S1 in File S1 for details). The last two covariates, 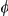 and 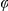 are used to model the effects of aspect and slope on growth, following methods outlined in following Clark (1990), where
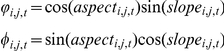
This representation is linear in aspect parameters, thus meeting assumptions of standard regression. If the slope is equal to zero, then aspect has no effect on growth, and slope magnifies the effect of aspect. The hypothesis of no effect of aspect corresponds to *β_5_ = β_6_ = 0*. For validation we compared observed and predicted diameters (also in the Supplemental material).

### Modeling mortality

The mortality data consist of a record of status (alive, dead) for individuals at the second and third census, denoted *s_i,j_*_,*t*_. We model this as *s_i_*_,*j*,*t*_ ∼ Bernoulli(*δ_i,j,t_* Δ*_i,j,t_*,) where *δ_i,j,t_* is the probability of survival for individual *i* in plot *j* over one year, and Δ*_i,j,t_* is the time interval. Initial exploration of the data indicated that the probability of survival increases with diameter for small trees, but for some species mortality declines with diameter for large trees. We therefore defined a linear predictor for the logit of *δ_i,j,t_* that includes an intercept, slope of diameter and slope of diameter-squared to capture the U-shape of mortality observed for many tree species (e.g., [32], [33]). As mortality was rare, other environmental covariates could not be fitted explicitly at this stage. Light, an important variable in plant survival, is captured by the diameter in the model (as diameter is strongly associated with canopy light status). Environmental covariates were related to survival in the higher-level species model (see below). The diameter measurement used was the posterior mean obtained from the growth model, i.e. diameter corrected for observation error. We used the ‘optim’ function in *R* to identify parameter values that maximized the Bernouilli likelihood (see Supplement).

### Inferring demographic rates in different environmental conditions

In order to explore more detailed ecological questions about trade-offs across species, we constructed predictive distributions for combinations of environmental conditions (slope, stand age, and light environment) and for two tree sizes: “small”, corresponding to twice the smallest diameter observed in the data-set (5 cm) and “large” corresponding to a diameter of 30 cm. We also predicted survival for large and small trees separately.

### Building a model with interactions between traits, ontogeny, and environment to predict vital rates

After arcsine transforming survival probabilities and taking the logarithm of seed mass to improve inference and to normalize variables, all variables were standardized to means of zero and variance of two standard deviations to enable ease of interpreting interaction terms, and ensure that binary variables, such as slope, forest age, and tree size, retain a unit range [34]. For vital rate variables we use the mean posterior values estimated from the Bayesian models. This averages over parameter uncertainty, but allows efficient model estimation and selection in a maximum likelihood setting. We then fit a series of linear regressions of increasing complexity including: i) univariate regressions of growth against survival, and growth and survival against all functional traits (independently); ii) extensions of these univariate regressions to include environmental variables and tree size, with separate ANCOVAs for each functional trait and with each environmental/ontogenetic variable fit as a factor; and iii) a multiple regression with growth regressed on all variables.

## Results

Medians and credible intervals for the nine demographic parameters estimated using the hierarchical Bayesian model describing growth for each of the original 41 species (see Appendix S2 in File S1, Table S1 in File S1, Fig. S1 for diagnostics) showed well-identified posterior distributions and accorded well with known demographic patterns (e.g., light substantially increased growth). For six species, credible intervals for at least one of the aspect posteriors did not include zero, indicating an effect of aspect on growth (Clark 1990) (starred column in Table S1 in File S1). In subsequent regression analyses, ‘slope’ is a bivariate variable with two values corresponding to either no slope or a slope of 0.5 and a northeast aspect.

**Figure 1:**
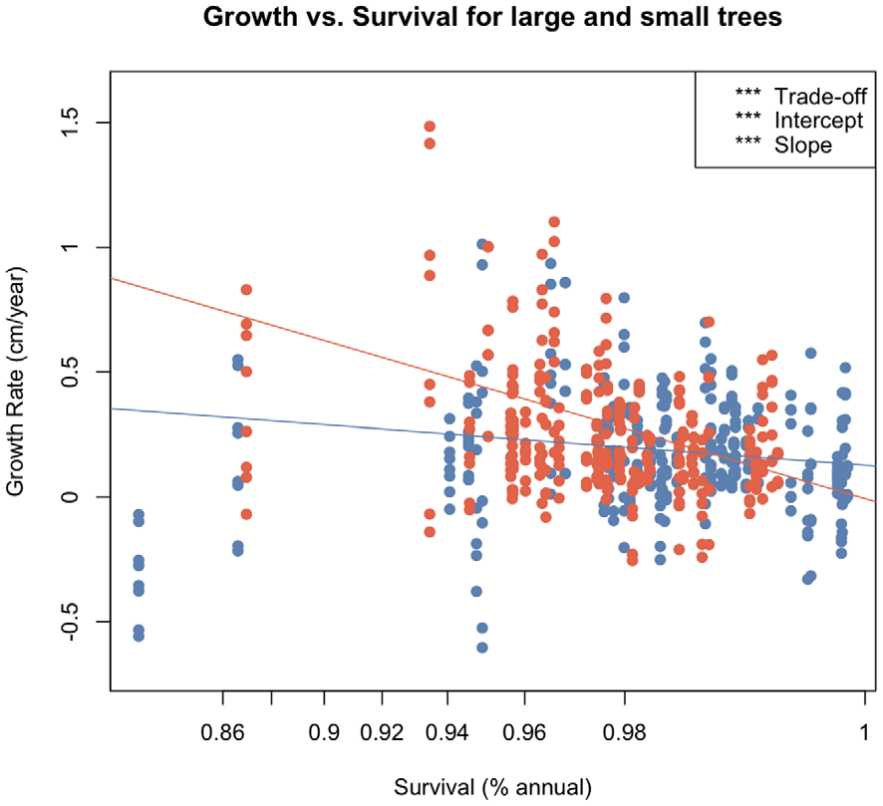
Growth versus survival for large trees (blue) and small trees (red). “Trade-off” shows an overall significant negative relationship between growth and survival. The ‘intercept’ term refers to a difference in intercepts between small and large trees, and a ‘slope’ difference refers to the different slope between large and small trees. These differences indicate that the growth survival trade-off is more pronounced for smaller trees (red line). ANOVA table details in Table 1. Significance codes for all figures: 0 ‘***’ 0.001 ‘**’ 0.01 ‘*’ 0.05 ‘.’ 0.1 ‘ ’ 1.

**Figure 2:**
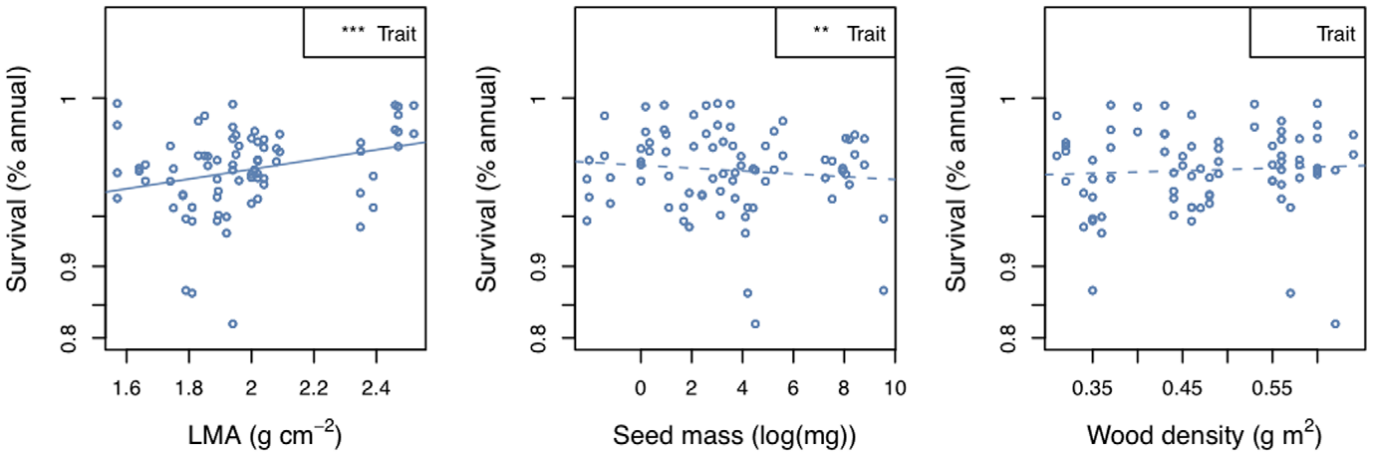
Survival versus functional traits. Leaf mass per area (LMA) shows a positive slope, indicating that species with thicker leaves tend to survive more, as predicted by theory. Seed mass and wood density show weak or no relationship to survival. ANOVA table details in Table 1. Significance codes for all figures: 0 ‘***’ 0.001 ‘**’ 0.01 ‘*’ 0.05 ‘.’ 0.1 ‘ ’ 1.

**Figure 3:**
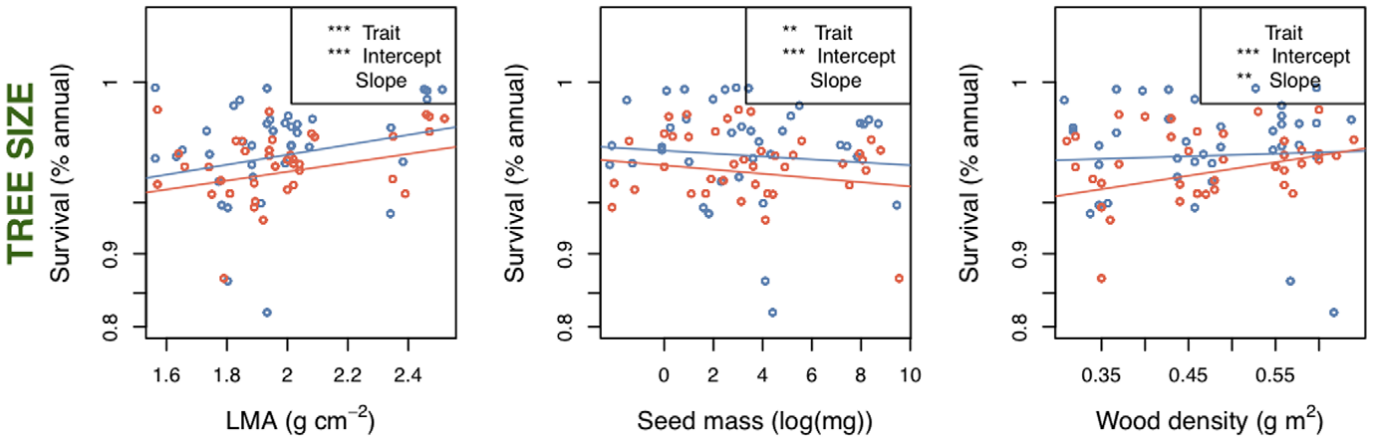
Survival versus functional traits with ontogenetic variable as a covariate. LMA and seed mass shows a difference between intercepts, while wood density shows no overall slope (see Figure 2 panel 3), but the interaction term shows a differential survival between large and small trees depending on wood density, with smaller trees showing an effect of wood density. Slope for large trees is blue and for small trees red. ANOVA table details in Table 1. Significance codes for all figures: 0 ‘***’ 0.001 ‘**’ 0.01 ‘*’ 0.05 ‘.’ 0.1 ‘ ’ 1.

**Figure 4:**
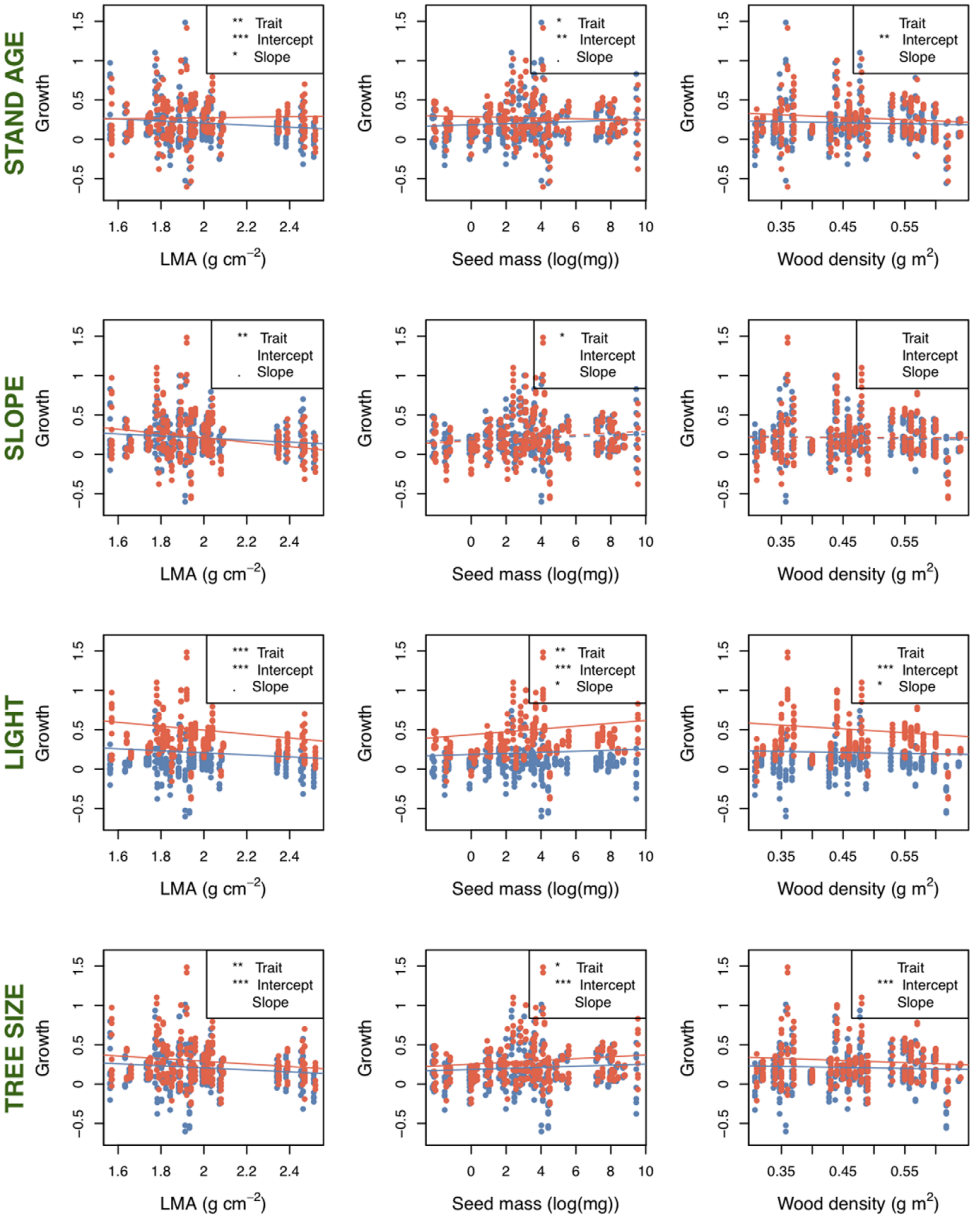
Growth versus functional traits with environmental and ontogenetic variables as covariates. Red slopes indicate old stands, plots on slopes, high light, and small trees, while blue indicates young stands, no slope, low light, and large tree levels. Light is the most important effect in these analyses, showing a strong positive shift to higher growth in high-light, regardless of the relationship between the traits and growth. There remains a great deal of scatter around these relationships, however, suggesting a need to test the variables and their interactions in a combined model (Table 2). ANOVA table details in Table 1. Significance codes for all figures: 0 ‘***’ 0.001 ‘**’ 0.01 ‘*’ 0.05 ‘.’ 0.1 ‘ ’ 1.

In the single variable regressions, growth against survival was negative (F_1, 622_ = 7.63, P = 0.006; r-squared = 0.078 [all r-squared values are ‘adjusted’ values, taking into account model complexity]) (Figure 1, Table 1) There were two series of models run with survival as a response variable: regressions with the different trait values (Figure 2), and an ANCOVA with tree size (large vs. small) as a covariate (Figure 3). ANCOVA results for growth are shown in Figure 4. In figure legends, “Trait” indicates the significance of the functional trait in the regression for either growth or survival given the effect of the context variable (environmental or ontogenetic), while “Intercept” and “Slope” indicate the significance of the context variable on the intercept or slope of the trait variable regression. Solid lines indicate overall regression significance (from the F statistic in the ANOVA table), while dashed lines indicate non-significant regression equations. All detailed results can be found in Table 1. Of the trait regressions, LMA showed a significant, positive influence on the species' annual survival estimates. Seed mass showed a statistical, but not ecological relationship to survival. Survival against wood density was not significant. When these regressions were re-run with tree size as a covariate (Figure 3), more interesting interactions were found. The slopes of the overall relationship remained the same as in Figure 2, but for LMA and seed mass the intercept varied between large and small trees. That is, in both cases, large trees survive better than small trees, but the relationship between traits and survival remains the same. For wood density, large trees showed no relationship with survival, but small trees showed the expected positive response, so that small trees with higher wood density survived more on average than low wood density species.

**Table 1:** Individual regressions and ANCOVAs.

Response	Covariate	Effect	F-value	P-value	Adjusted R-squared	Figure reference
Growth	Survival		7.63	**0.0060**	0.08	1
Growth	LMA	Stand Age	9.93	**<0.0001**	0.04	4
Growth	Seed mass	Stand Age	6.92	**<0.001**	0.03	4
Growth	Wood density	Stand Age	4.84	**0.003**	0.02	4
Growth	LMA	Slope	5.46	**<0.001**	0.02	4
Growth	Seed mass	Slope	2.33	0.096	0.01	4
Growth	Wood density	Slope	0.50	0.690	0.00	4
Growth	LMA	Light	126.00	**<0.0001**	0.39	4
Growth	Seed mass	Light	120.00	**<0.0001**	0.38	4
Growth	Wood density	Light	116.00	**<0.0001**	0.37	4
Growth	LMA	Tree Size	9.27	**<0.0001**	0.04	4
Growth	Seed mass	Tree Size	6.97	**<0.001**	0.03	4
Growth	Wood density	Tree Size	5.47	**<0.01**	0.02	4
Survival	LMA	Stand Age	18.00	**<0.0001**	0.12	3
Survival	Seed mass	Stand Age	0.00	0.1210	0.00	3
Survival	Wood density	Stand Age	5.91	**<0.001**	0.02	3
Survival	LMA		56.30	**<0.0001**	0.08	2
Survival	Seed mass		7.20	**<0.01**	0.00	2
Survival	Wood density		2.15	0.1430	0.02	2

Degrees of freedom for the bivariate regressions were 2 and 590, and for the ANCOVAs, 3 and 588. Bold values significant.

Growth showed varying relationships with traits and the binary covariates. Overall regression relationships between growth and the functional trait values followed a pattern similar to that of survival, with LMA showing the strongest relationship, seed mass a week but significant relationship, and wood density showing no relationship (Figure 4, Table 1).

**Figure 5:**
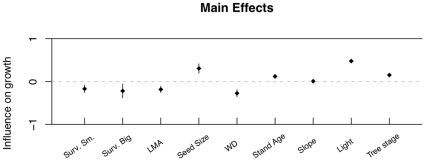
Main effects of the combined model. Because the variables were standardized using two standard deviations, the main effects of the combined model (Table 2) show standard deviation changes in growth given standard deviation changes in predictor variables, given that all other variables are held at the mean values across all species and conditions. Bars show standard errors of parameter coefficients. Light shows the largest positive influence, but other variables, through their interactions, also affect growth (Table 2).

For the combined regression model of growth against all functional traits, environment/ontogenetic variables, and survival estimates, the optimal model selected by the stepAIC function in R [35] contained 30 coefficients (Table 2). The R-square for this model was 0.66. AIC scores for comparing best first-, second-, and third-order models were 1509, 1716, and 1738 respectively (full results can be found in Appendix S2 in File S1). The main effects from this model show that light and seed size show the greatest positive influence on growth, while wood density the greatest negative effect (Figure 5, Table 2).

**Table 2:** Optimal regression model of species growth rates against survival and ontogenetic, environmental, and trait variables.

Coefficients:	Estimate	Std. Error	t value	Pr(>|t|)	
(Intercept)	0.11267	0.01674	6.73	4.00E-11	***
survsm	−0.16849	0.04439	−3.8	1.60E-04	***
survbig	−0.21941	0.08115	−2.7	7.06E-03	**
LMA	−0.18358	0.03696	−4.97	8.90E-07	***
SeedSize	0.30533	0.05517	5.53	4.70E-08	***
WD	−0.27357	0.04192	−6.53	1.50E-10	***
stand_age	0.12056	0.02101	5.74	1.50E-08	***
slope	0.00823	0.01945	0.42	6.72E-01	
light	0.47762	0.01949	24.51	2.00E-16	***
is_small	0.15139	0.02018	7.5	2.30E-13	***
survsm:light	−0.25103	0.04359	−5.76	1.40E-08	***
survsm:WD	−0.52458	0.07362	−7.13	3.00E-12	***
survbig:WD	1.60038	0.18138	8.82	2.00E-16	***
survbig:LMA	1.27205	0.26909	4.73	2.80E-06	***
survsm:LMA	−0.50532	0.12538	−4.03	6.30E-05	***
survbig:is_small	−0.30681	0.0747	−4.11	4.60E-05	***
LMA:stand_age	0.16952	0.04847	3.5	5.00E-04	***
survsm:slope	−0.10386	0.04215	−2.46	1.40E-02	*
SeedSize:stand_age	−0.06204	0.0421	−1.47	1.41E-01	
LMA:slope	−0.08168	0.04099	−1.99	4.68E-02	*
SeedSize:light	0.15198	0.05032	3.02	2.64E-03	**
WD:light	−0.14239	0.05208	−2.73	6.44E-03	**
survsm:SeedSize	0.34885	0.1104	3.16	1.66E-03	**
SeedSize:WD	−0.13973	0.05405	−2.59	9.97E-03	**
survbig:SeedSize	−1.37951	0.39661	−3.48	5.40E-04	***
LMA:SeedSize	0.31982	0.09057	3.53	4.50E-04	***
SeedSize:is_small	0.11278	0.04363	2.59	9.97E-03	**
survbig:LMA:SeedSize	−1.54015	0.46632	−3.3	1.01E-03	**
LMA:SeedSize:stand_age	0.27	0.126	2.14	3.25E-02	*
survbig:SeedSize:is_small	−0.88274	0.22562	−3.91	1.00E-04	***

Using stepwise model selection on AIC scores, this 3rd order interaction model was selected as the minimum adequate model. ANOVA table statistics: F_29, 594_ = 40.1, P<0.0001, R-square = 0.64. Ontogenetic variable ‘is_small’ is 0 for small trees and 1 for large trees.

Signif. codes: 0 ‘***’ 0.001 ‘**’ 0.01 ‘*’ 0.05 ‘.’ 0.1 ‘ ’ 1.

## Discussion

All of the analyses in this study support a basic trend of a growth-survival trade-off among these temperate forest tree species (Figure 1), in line with an important hypothesized mechanism of tree species coexistence [14]. As has been shown in other studies (e.g., [6]), we also found that species' functional traits, specifically LMA and seed mass, are associated with both growth and survival in bivariate regressions, providing a potential physiological mechanism to explain this trade-off. However, this study extends these links between functional traits and demographic vital rates to show that environmental and ontogenetic contexts in which these relationships occur are critical to evaluating how tree species with different physiological traits respond differently under different conditions. The size class of the trees, the light environment, and features of the stand, such as age, were all important covariates in explaining variation in growth, and the negative relationship between growth and survival.

Light showed the strongest effects in the regressions (Table 1), and not only best predicted growth, but interacted with the survival term, indicating an adjustment in the trade-off between growth and survival, depending on the light-levels experienced by the tree. This follows research showing that while many species can grow and survive in high-light, low-light environments distinguish shade-tolerant from shade-intolerant species [12]. In the ANCOVA models (Figures 3 and 4), differences attributable to environmental covariates may swamp the effect of functional traits (non-significant trait slopes in Figure 4); but in some cases changes in the slope are detected. We found trends for a change in slope of growth against seed mass and wood density under high and low light conditions. That is, how species' traits predict growth changes depending on the level of light, as predicted by theory of niche partitioning [11]. We found that the relationship between LMA and growth showed a trend of change in young versus old-age forest stands.

Overall, however, the clearest patterns of context dependence related to survival and tree size, clearly showing a difference in responses between small and large trees. This demonstrates the potential critical relevance of ontogeny in trade-offs that can lead to coexistence. In the overall regression of growth versus survival, the negative slope is significantly steeper in small vs. large trees (Figure 1), indicating a stronger trade-off for small trees. The relationship between functional traits and growth and survival also changes when the size of the tree is taken into account. For example, the relationship between LMA and survival switches signs for small and large trees (Table 2). The same reversal of effect is true with wood density and seed size. Thus, the allocation of materials to traits as a strategy, may be important for different tree species at different times in their life-histories. This is an important point to remember when testing theories of trait allocation and vital rates, as focusing on a narrow life-history range (e.g., saplings or canopy adults), may affect the power, and even the direction of inference. Kraft et al. [5] found that the trait distribution of species in the Amazon understory shifted when comparing saplings to canopy adults.

**Figure 6:**
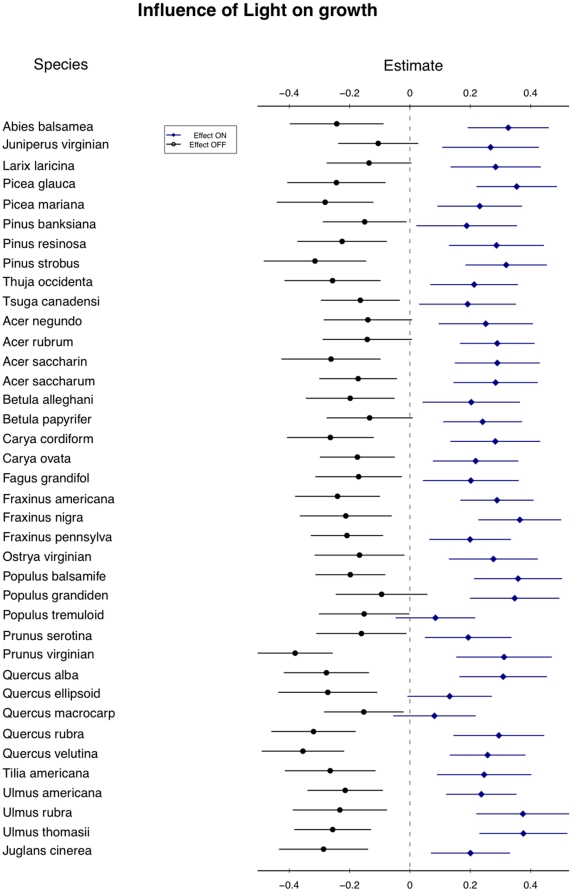
Influence of light on predicted growth across species. This figure shows the difference in expected growth due to high and low light across species. All other variables are kept at their means (zero). Lines show expected growth over two standard errors of parameter values. Regardless of expected average growth rate, light increases growth across all species.

**Figure 7:**
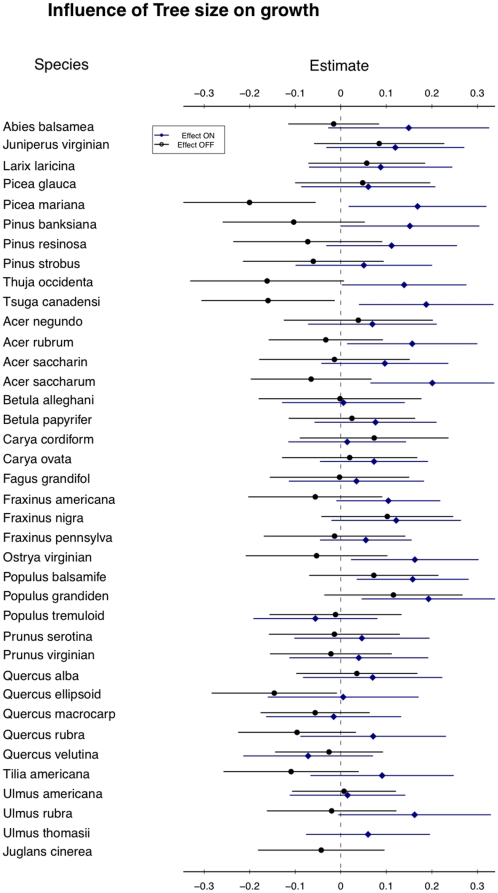
Influence of tree size on growth rate. The change in expected growth due to ontogeny shows a range of effects across species. All other variables are kept at their means (zero). Lines show expected growth over two standard errors of parameter values. Some species are far more sensitive to ontogenetic stage in growth ability. Combining this information with Figure 6 shows even focusing on two effects can begin to divide niche space across species, with high-light, low-light, short and tall trees differentially affecting growth.

The interactions demonstrated here between traits, demographic rates, and the environment indicate how fine-scale partitioning of environmental gradients (stand age, aspect, etc.) can result in local niche dimensions that can explain species coexistence. This approach to coexistence has its roots not in the low-dimensional niche explanation of coexistence (e.g., [36]), but in the high-dimensional niche space of Hutchinson [24] or MacArthur [25]. Low-dimensional niche coexistence is supported when a one or a few fundamental resources, such as light or nitrogen, can be partitioned across a gradient by species who do better than their nearest competitors in one location of the gradient. In this study, no single resource is likely to provide the fine gradient that 41 species can differentially exploit. Light, the most important resource for growth in this study (Figure 5), is equally important to all species and gives similar shifts in growth response (Figure 6). As growth is generally more important for smaller trees than larger trees (Figure 1), the increased growth in high light (Figure 4) increases the importance of the ontogenetic trade-off. This interaction is itself different for different species. Although light is uniformly beneficial to growth across all species, the effect of tree size on growth is different for different species (Figure 7). Some species show a large shift in growth effect when large versus when small (e.g., *Picea mariana*), others show little effect (e.g., *Juniperus virginian*), while other species show a trend towards a reversal of the size-growth relationship (e.g., *Carya cordiform* and *Quercus vlutina*). Such high-dimensional niche space reflects many potential axes along which species can avoid competition through differential resource exploitation.

The importance of interactions in this analysis highlights the difficulty in determining relationships between fitness and traits without including ontogeny and environmental context in an analysis. Analyses that ignore interactions may show no effect or only loose effects (e.g., the spread of points in two-dimensional models of Figures 2, 3 and 4), when in higher dimensions there exist effects of opposite sign that cancel one another out or obscure the relationship. For example, wood density is not a significant predictor of survival in the univariate regression, but is when interacting with tree size. Two-dimensional graphs of trade-offs may be helpful conceptually, but the important interactions that demonstrate niche-based coexistence can only be understood through building models that cannot be portrayed in a simple figure. Failing to investigate the conditional dependencies between vital rates, ontogeny, traits, and local habitat can make species with known ecological differences appear equivalently fit. Ontogeny, especially, seems important to the vital rates of these tree species, and yet the role of changing rates over life-history, and how the environment influences those relationships requires more focused theoretical, observational, and experimental research. For long-lived species like trees, especially, this poses a challenging but potentially important future focus. Because these results show that smaller trees are generally more sensitive to the trait-mediated trade-offs, studies of forest biodiversity change under future climate scenarios need to focus on the entire life-history of the trees, as adults may tolerate climate shifts, but succeeding generations show vulnerability to those changes.

### Conclusions

Although a changing environment will likely affect many species in temperate forests, the direction and strength of those influences needs to be modeled on a species by species basis as we show that interactions are critical to interpreting demographic patterns, and by extension population dynamics. These responses can then be collected into predictions of forest community response to climate through simulations. Analyses that fail to incorporate a suite of important mechanisms that affect vital rates will fail to predict accurately how species might respond to environmental shifts, and also fail to scale up to community dynamics. Advancing our understanding of the mechanisms that guide tree species coexistence will require detailed and context-specific measurement of functional traits, and intensive monitoring of local environmental conditions across tree-life history.

## Supporting Information

Figure S1Observed (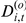) and predicted diameter accounting for measurement error and the process model for growth (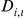) for *Larix laricinia* from the posteriors of the Gibbs sampler described above. Points that are far above the line correspond to situations where the previous size was very large, since the minimum increment was set to 0.001 these could not be attained within the confines of the process model; points that are far below the line correspond to situations where the previous size was very small, since the maximum increment was set to 7 cm these could not be attained within the confines of the process model.(TIFF)

File S1 **Contains the following supporting files:** Appendix S1: The Gibbs sampler for growth and model for survival; Appendix S2: Phylogenetically independent contrast tests; Table S1: Mean p-value associated with the slope of a regression relating Phylogenetically Independent Contrasts for demographic rates (rows) against functional traits (columns); bold indicates slopes significant at the 0.05 level, underlined at the 0.10 level; Table S2: Mean coefficients associated with Table S1 in File S1, with significant values at the 0.05 level shown in bold, and at the 0.1 level underlined.
(DOC)
