# Supplementary figures and images for: High-Dimensional Coexistence of Temperate Tree Species: Functional Traits, Demographic Rates, Life-History Stages, and Their Physical Context
Source: PLoS One. 2011 Jan 31;6(1):e16253. doi: 10.1371/journal.pone.0016253 (PMC3031558; doi:10.1371/journal.pone.0016253)

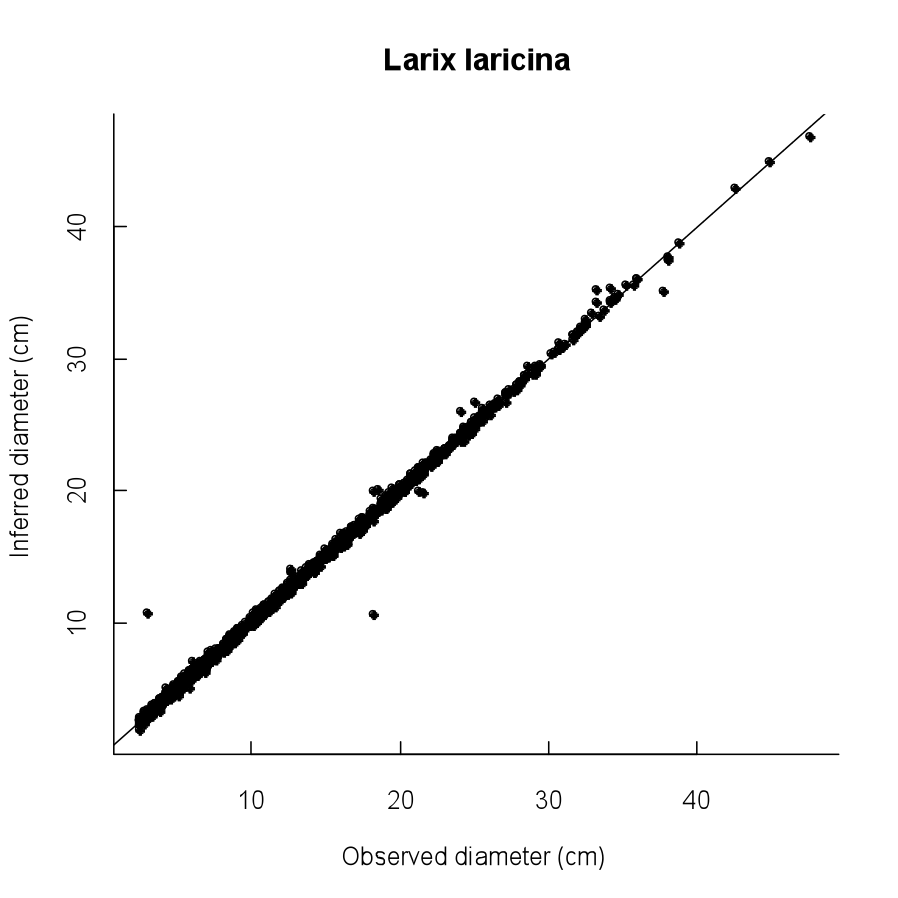

Supplement: Figure S1 — Observed () and predicted diameter accounting for measurement error and the process model for growth () for Larix laricinia from the posteriors of the Gibbs sampler described above. Points that are far above the line correspond to situations where the previous size was very large, since the minimum increment was set to 0.001 these could not be attained within the confines of the process model; points that are far below the line correspond to situations where the previous size was very small, since the maximum increment was set to 7 cm these could not be attained within the confines of the process model. (TIFF) [file pone.0016253.s001.tiff]
