# Supplementary material for: High-Dimensional Coexistence of Temperate Tree Species: Functional Traits, Demographic Rates, Life-History Stages, and Their Physical Context
Source: PLoS One. 2011 Jan 31;6(1):e16253. doi: 10.1371/journal.pone.0016253 (PMC3031558; doi:10.1371/journal.pone.0016253)
Supplement: File S1 — Contains the following supporting files: Appendix S1: The Gibbs sampler for growth and model for survival; Appendix S2: Phylogenetically independent contrast tests; Table S1: Mean p-value associated with the slope of a regression relating Phylogenetically Independent Contrasts for demographic rates (rows) against functional traits (columns); bold indicates slopes significant at the 0.05 level, underlined at the 0.10 level; Table S2: Mean coefficients associated with Table S1 in File S1, with significant values at the 0.05 level shown in bold, and at the 0.1 level underlined. (DOC) [file pone.0016253.s002.doc]

**Appendix S1: Bayesian Models of Demographic Rates**

*Modeling uncertainty in diameter measurements*

A first challenge to modeling this dataset is that there may be errors in measurements leading to unlikely large negative growth increments. To allow for measurement error, as well as shrink and swell of the bole, we define a model to describe error in the measured diameter values. We assume that the observed diameter in cm of individual *i* at time *t* in plot *j*, $D_{i,j,t}^{(o)}$, (where “observation” is indicated by the subscript (*^o^*)) is normally distributed around the “true” diameter with a variance, *w* that captures measurement error, e.g., $D_{i,j,t}^{(o)}\sim N(D_{i,j,t},w)$.

We will fit the growth models (and mortality models) to this distribution of “true” diameter values.

*Modeling growth with uneven census intervals*

The next challenge to inferring yearly growth increments in this dataset is that census intervals are uneven. Simply estimating yearly growth by dividing the multi-annual growth increment by the time interval between censuses will underestimate uncertainty in growth rates, because variance accumulates over intervening years. For example, after 10 years, growth increments will be more variable than after just 5 years. We can avoid this bias by using a Bayesian modeling approach to estimate variance accumulation in growth. Denoting *D**_i_*_,j_,*_t_* as diameter in cm of individual *i* at time *t* in plot *j*, and Δ the time between censuses, we define the increment between diameter at *t* and diameter at *t*+Δ by $\Delta d_{i,j,t}=D_{i,j,t+\Delta}-D_{i,j,t}$. If the mean growth increment in one year for individual *i* in plot *j* in year *t* is $\mu_{i,j,t}$, and the variance of such yearly growth increments is *σ*^2^, then

.

(1)

Multiplying the variance by Δ, the time between censuses, captures a standard assumption of white noise in yearly increments.

Having established the density function used for modeling growth (1), we need to define covariates and associated parameters we wish to estimate for the growth process. We modeled the mean structure *μ* as

, (2)

where the *i* index refers to individual, the *j* index refers to the plot number, and the *t* index refers to the time step, *D* is diameter in cm, *L* is the light index, *A* is estimated stand age, ***β*** is a vector of parameters, *a_i_* is a random individual effect, and *b_j_* is a random plot effect. The individual effect value accounts for variation over and above that defined by covariates, and that remains consistent during individual’s lifetime (Clark *et al.* 2003). Individual effects are distributed according to ***a****~N*(*0, τ^2^*). Likewise the plot effect accounts for variation over and above that defined by covariates (including consistent individual differences) that can be attributed to consistent plot differences, and plot effects are distributed according to ***b****~N*(*0, κ^2^*).

The last two covariates, $\varphi$ and $\phi$ are used to model the effects of aspect and slope on growth, following methods outlined in following Clark (1990), where

$$\begin{matrix} \varphi_{i,j,t}=\cos(aspect_{i,j,t})\sin(slope_{i,j,t}) \\ \phi_{i,j,t}=\sin(aspect_{i,j,t})\sin(slope_{i,j,t}) \end{matrix}$$

This representation is linear in aspect parameters, thus meeting assumptions of standard regression. If the slope is equal to zero, then aspect has no effect on growth, and slope magnifies the effect of aspect. The hypothesis of no effect of aspect corresponds to *β_5_= β_6_=0*.

*The full probability density function for growth: model and priors*

The full conditional density function for the diameter latent state variable for one tree year, which includes both the observation error model and the growth process model outlined above is

$=N\left(D_{i,j,t+\Delta}^{(o)}\left|D_{i,j,t+\Delta},w\right.\right)$ $N\left(D_{i,j,t+\Delta t}-D_{i,j,t}\left|\Delta_{i,j,t}\mu_{i,j,t},\Delta_{i,j,t}\sigma^{2}\right.\right)$.

To obtain the full model, we multiply this by the probability density functions associated with the random effects, *N*(*a_i_*|0*, τ^2^*), and *N*(*b_j_*|0*, κ^2^*) and the prior probability density functions assigned to all parameters. Specifically, we set a normally distributed prior on the ***β*** parameters, e.g.,$\beta\sim N(\beta_{0},V_{\beta})$ where ***β****_0_* is a vector of means for the multivariate prior distribution, and ***V_β_*** is the corresponding variance-covariance matrix. We set an inverse gamma prior on the process error variance, , where and *α* and *γ* are parameters defining the prior mean and variance of the error. We set inverse gamma priors on the other variance parameters , , and $w\sim IG(w_{1},w_{2})$ likewise.

The prior mean vector for the ***β*** parameters, ***β****_0_*, was set to zero, and the variance for each parameter was set to 100. This corresponds to an uninformative prior. We used an informative prior for the observation error variance, *w*, with a mean of 0.15 based on previous results (Clark *et al.* 2007) and a small variance. The magnitude of the variance was set so that it weights the prior mean by twice the number of observations for each species, *n_s_*, (through moment matching, Clark 2007), e.g.,

$$\begin{matrix} w_{1}=2n_{s} \\ w_{2}=0.15(w_{1}-1) \end{matrix}$$

For individual variance, we used a large prior mean of 0.4, and weighted it weakly by two (most species had at least 30 individuals), making the prior uninformative. For process error, *σ^2^* we use a large prior mean of 0.5, weighted by 10 for all species, which again is uninformative, since in most cases the number of observations per species is greater than 100.

*Gibbs sampling*

Gibbs sampling is a Markov Chain Monte Carlo (MCMC) technique that involves alternate sampling from each of the full conditional distributions of all unknowns in the model, including parameters and latent variables (Clark, 2007, Gelfand & Smith, 1990). Taking the full conditional of the distribution introduced above, we have

$p\left(D_{i,j,t+\Delta}^{(o)},D_{i,t+\Delta}|D_{i,j,t+\Delta}^{(o)},\beta,\alpha,w,\sigma^{2},\tau^{2},\kappa^{2}\right)\propto N\left(D_{i,j,t+\Delta}^{(o)}\left|D_{i,j,t+\Delta}\right.,w\right)$ $N\left(D_{i,j,t+\Delta t}\left|D_{i,j,t}+\Delta_{i,j,t}\mu_{i,j,t},\Delta_{i,j,t}\sigma^{2}\right.\right)$.

A Metropolis sample from this distribution begins with a proposal for the first diameter value from a normal density centered on the first observation, $D_{i,j,1}^{(o)}$. Then all diameter increments for individuals alive are proposed from a normal distribution truncated between 0.001 and 7 to ensure that diameter increments are never implausibly large or small. Proposals for diameters for every individual at every time-step are obtained as the sum of the proposal for the first diameter and the cumulative sum of growth increments. Proposals are accepted on a tree by tree basis using the likelihood above, with the second density function on the right hand side, i.e., the conditional distribution of average growth increments taken to be, for an individual *i*,

$$p\left(\Delta d_{i,j}|D_{i,j}^{(o)},\beta,\alpha,w,\sigma^{2},\tau^{2},\kappa^{2}\right)\propto\prod_{1}^{3} N(\Delta d_{i,j,t}|V_{i,j,t}v_{i,j,t},V_{i,j,t})N(D_{i,j,t}^{(o)}|D_{i,j,t},w)I(0.001\Delta_{i,t},7\Delta_{i,t})$$

where *I* is the indicator function and insures that growth increments are contained between a chosen lower bound of 0.001 cm y^-1^ and upper bound of 7 cm y^-1^ and

$$\begin{matrix} V_{i,t}^{-1}=1/[\Delta_{i,t}\sigma^{2}] \\ v_{i,t}=\Delta_{i,t}\mu_{i,t}/[\Delta_{i,t}\sigma^{2}]=\mu_{i,t}/\sigma^{2} \end{matrix}$$

The next step is to update the parameters. The process model above combined with the chosen prior distributions develop out to provide conditional distributions for the *σ*^2^, *w*, *κ*, *τ*, *a_i_*, *b_j_* and ***β*** parameters that can be directly sampled. For the ***β*** parameters, taking $N(\Delta d_{i,j,t}|\Delta_{i,j,t}\mu_{i,j,t},\Delta_{i,j,t}\sigma^{2})N(\beta|\beta_{0},V_{\beta})$, and “incompleting the square” (Clark, 2007), we have

$V^{-1}=\sum\frac{Z(X_{\Delta=Z}^{T}X_{\Delta=Z})}{\sigma^{2}}+V_{0}^{-1}$ and

$v=\sum\frac{X_{\Delta=Z}^{T}(\Delta d_{\Delta=Z}-Za_{\Delta=Z}-Zb_{\Delta=Z})}{\sigma^{2}}+\frac{\beta_{0}}{V_{0}}$,

where *X*_Δ=_*_Z_* is the matrix of covariates for all individuals *i* at all times *t* where the time between censuses Δ=*Z*, *d*_Δ=_*_Z_*, *a*_Δ=_*_Z_* and *b*_Δ=_*_Z_* are defined likewise, and the sum in each case is taken over all possible values for *Z*, and the superscript *^T^* indicates the matrix transpose operation. With this, we can directly sample the *β* parameters, $\beta\sim N(Vv,V)$. To facilitate convergence, we constrained the slopes of log diameter and log diameter squared to be negative by sampling from truncated normal distributions. For the individual deviations, we can define,

$V_{i}^{-1}=\sum_{t=1}^{T_{i}} \frac{\Delta_{i,j,t}}{\sigma^{2}}+\frac{1}{\tau^{2}}$ and

,

where *T_i_* indicates the maximum number of time increments available for individual *i*, other variables as above. The individual growth components can then be directly sampled from $a_{i}\sim N(V_{i}v_{i},V_{i})$. Likewise, for the plot deviation we can define,

$V_{j}^{-1}=\sum_{t=1}^{T_{i}} \frac{\Delta_{i,j,t}}{\sigma^{2}}+\frac{1}{\kappa^{2}}$ and

$$v_{i}=\sum_{t=1}^{T_{i}} \frac{(\Delta d_{i,j,t}-\Delta_{i,j,t}X_{i,j,t}\beta-\Delta_{i,j,t}b_{j})}{\sigma^{2}}$$

and plot intercepts can be directly sampled from $b_{j}\sim N(V_{j}v_{j},V_{j})$.

For the variance parameters,

$$\sigma^{2}\sim IG\left(\alpha+\frac{TN}{2},\gamma+0.5\sum_{\Omega} \frac{\left(\Delta d_{i,t}-\Delta_{i,t}X_{i,t}\beta-\Delta_{i,t}a_{i}\right)^{2}}{\Delta_{i,t}}\right)$$

where *Ω* indicates all available combinations of *i* and *t*,

$$\tau^{2}\sim IG\left(\alpha i+N,\gamma i+0.5\sum_{i} a_{i}^{2}\right)$$

and

$$\kappa^{2}\sim IG\left(\alpha j+N,\gamma j+0.5\sum_{i} b_{j}^{2}\right)$$

We checked plots of parameter estimates over iterations to verify for convergence and compared the observed and predicted latent diameters to check for systematic biases (Fig. S1).

*Modeling mortality with uneven census intervals*

The mortality data consists of a record of status (alive, dead) for individuals at the second and third census, denoted *s_i,j_*,*_t_*. We model this as

*s_i_*,*_j_*,*_t_* ~ Bernoulli($\delta_{i,\text{j,}t}^{\Delta i,\text{j,}t}$),

where *δ_i,j,t_* is the probability of survival for individual *i* in plot *j* over one year, and Δ*_i,j,t_* is the time interval. Initial exploration of the data indicated that the probability of survival increases with diameter for small trees, but for some species mortality declines with diameter for large trees. We therefore defined a linear predictor for the logit of *δ_i,j,t_*

$\log it(\delta_{i,j,t})=m_{S}+m_{S,D}\log D_{i,j,t}-m_{S,D2}(\log D_{i,j,t})^{2}$,

where *m_s_* indicates the species specific intercept, *m_s,D_* the species specific slope of diameter, and *m_s,D2_* the species specific slope of diameter squared, to capture the U-shape of mortality observed for many tree species (e.g., Monserud & Serba, 1999, Platt *et al.* 2002). The diameter measurement used was the posterior mean obtained from the growth model. For simplicity, we used a constrained maximum likelihood approach to obtain parameter estimates, with the *m_s,D_* parameter constrained to be positive and the *m_s,D2_* parameter constrained to be negative. For model validation, we compared average and predicted survival across chosen size bins. As for growth, size will change over the time interval *Δ*, so that the linear predictor will not capture dynamics during the interval. However, the change is very small relative to the range of the data.

**References**

Clark, J.S. (2007) *Models for Ecological Data.* Princeton University Press.

Gelfand, A.E. & Smith, A.F.M. 1990. Sampling-based approaches to calculating marginal densities. *Journal of the American Statistical* *Association* 85:398–409.

**Appendix S2: Phylogenetically Independent Contrasts for the effects of functional traits on demographic rates.**

We obtained a phylogeny of the 39 species using http://www.phylodiversity.net/phylomatic/. To compare different posteriors means of demographic features and functional traits having removed the inherited component of variation, we generated phylogenetically independent contrasts (PICs) for standardized values using the Ape package in R (R Development Core, 2007). Branch lengths were not specified. Since the phylogeny available included multichotomies, for each comparison, we sampled over all possible trees obtained by resolving multichotomies into dichotomies, and took the mean slope of regressions forced through the origin, and the mean p-value (see Tables S1 and S2).

**Table S1: Mean p-value associated with the slope of a regression relating Phylogenetically Independent Contrasts for demographic rates (rows) against functional traits (columns); bold indicates slopes significant at the 0.05 level, underlined at the 0.10 level.**

|  | logLMA | logSeedSize | SpecificGrav |
| --- | --- | --- | --- |
| Litt.Light.Yg.NS | 0.78 | 0.69 | 0.05 |
| Litt.Light.Old.NS | 0.27 | 0.53 | 0.50 |
| Litt.Dark.Yg.NS | 0.31 | 0.30 | **0.04** |
| Litt.Dark.Old.NS | 0.06 | 0.53 | 0.21 |
| Litt.Light.Yg.Sl | 0.87 | 0.85 | **0.04** |
| Litt.Light.Old.Sl | 0.46 | 0.49 | 0.09 |
| Litt.Dark.Yg.Sl | 0.47 | 0.55 | 0.05 |
| Litt.Dark.Old.Sl | 0.40 | 0.93 | 0.10 |
| Big.Light.Yg.NS | 0.97 | 0.77 | 0.07 |
| Big.Light.Old.NS | 0.61 | 0.20 | 0.97 |
| Big.Dark.Yg.NS | 0.08 | 0.61 | **0.04** |
| Big.Dark.Old.NS | 0.11 | 0.44 | 0.95 |
| Big.Light.Yg.Sl | 0.95 | 0.75 | 0.05 |
| Big.Light.Old.Sl | 0.72 | 0.17 | 0.34 |
| Big.Dark.Yg.Sl | 0.28 | 0.85 | 0.06 |
| Big.Dark.Old.Sl | 0.24 | 0.48 | 0.30 |
| Small.surv | 0.71 | 0.92 | **0.00** |
| Big.surv | 0.73 | 0.73 | 0.91 |

**Table S2:** Mean coefficients associated with Table S1, with significant values at the 0.05 level shown in bold, and at the 0.1 level underlined

|  | logLMA | logSeedSize | SpecificGrav |
| --- | --- | --- | --- |
| Litt.Light.Yg.NS | 0.08 | 0.15 | -0.48 |
| Litt.Light.Old.NS | 0.35 | -0.27 | -0.19 |
| Litt.Dark.Yg.NS | -0.27 | 0.36 | **-0.46** |
| Litt.Dark.Old.NS | -0.46 | 0.21 | -0.28 |
| Litt.Light.Yg.Sl | 0.04 | 0.07 | **-0.48** |
| Litt.Light.Old.Sl | 0.20 | -0.25 | -0.39 |
| Litt.Dark.Yg.Sl | -0.19 | 0.21 | -0.44 |
| Litt.Dark.Old.Sl | -0.2 | 0.03 | -0.35 |
| Big.Light.Yg.NS | 0.01 | -0.1 | -0.43 |
| Big.Light.Old.NS | 0.14 | -0.45 | 0.01 |
| Big.Dark.Yg.NS | -0.45 | 0.18 | **-0.47** |
| Big.Dark.Old.NS | -0.40 | -0.26 | 0.01 |
| Big.Light.Yg.Sl | -0.02 | -0.11 | -0.46 |
| Big.Light.Old.Sl | 0.09 | -0.47 | -0.22 |
| Big.Dark.Yg.Sl | -0.28 | 0.07 | -0.43 |
| Big.Dark.Old.Sl | -0.29 | -0.23 | -0.23 |
| Small.surv | -0.09 | -0.03 | **0.60** |
| Big.surv | -0.07 | -0.10 | 0.02 |
